# Eosinophilic Granulomatosis With Polyangiitis Presenting With Visual Problems and Subendocardial Fibrosis, A Case Report

**DOI:** 10.1002/ccr3.70427

**Published:** 2025-04-17

**Authors:** Mahshid Talebi‐Taher, Sobhan Mehdipourrabori, Soroush Mostafavi, Mahboubeh Pazoki

**Affiliations:** ^1^ Infectious Diseases Department, Faculty of Medicine Iran University of Medical Sciences Tehran Iran; ^2^ Department of Cardiology, Hazrat‐e Rasool General Hospital, School of Medicine Iran University of Medical Sciences (IUMS) Tehran Iran

**Keywords:** EGPA, eosinophilic granulomatosis with polyangiitis, subendocardial fibrosis, vasculitis

## Abstract

This case highlights the atypical presentation of eosinophilic granulomatosis with polyangiitis (EGPA) with neurological and cardiac complications, emphasizing the necessity of early recognition and aggressive treatment to prevent morbidity and mortality.

## 1 Introduction

EGPA, formerly called Churg–Strauss syndrome, is a systemic necrotizing vasculitis of small‐ and medium‐sized vessels, characterized by asthma and blood eosinophilia. It usually occurs in individuals with preexisting asthma and involves the skin, lungs, and peripheral nerves [1, 2]. It exhibits a high prevalence among both females and males in their third and fourth decades of life [2].

Cardiac manifestations are reported in 30%–45% of patients, more common than other ANCA‐associated vasculitis (AAVs) [3]. All heart structures may be involved, but the main pattern is cardiomyopathy, resulting from endo‐myocardial eosinophilic infiltration [4].

EGPA usually affects the skin, heart, lung, and gastrointestinal tract. Ocular involvement in EGPA is unusual. It can manifest as conjunctival nodules, orbital myositis, orbital inflammatory syndrome, dacryoadenitis, and cranial nerve palsy [5, 6].

Ocular manifestations in EGPA are rare and include corneal ulceration, conjunctival granuloma, uveoscleritis, amaurosis fugax, orbital inflammatory pseudotumor, ischemic optic neuropathy, third and fourth cranial nerve palsy, and retinal artery occlusion [7, 8].

In this case report, we present a case of EGPA characterized by third and fourth cranial nerve palsy, cerebral embolic stroke, and subendocardial fibrosis.

## 2 Case History/Examination

A 45‐year‐old man was referred to the hospital with a history of asthma (the patient mentioned the history of taking salbutamol inhaler and antihistamine tablets, irregularly) presented with decreased vision, diplopia, and ptosis in the right eye that had persisted for 3 days. Systemic evaluation revealed no evidence of hypertension, diabetes, dyslipidemia, cardiovascular disease, or any other systemic disease. The patient stated that he had a history of right eye ptosis 10 years ago, and he received prednisolone for 10 days. After that, the symptoms resolved; a biopsy was taken from the patient's lacrimal gland, which was reported granuloma. He doesn't use tobacco, alcohol, or illicit drugs. He also had no history of intraocular surgery. Clinical examination showed blood pressure (BP), pulse rate (PR), and respiratory rate (RR) in the normal range. Visual acuity was 20/50 in the right eye and finger count at 3 m in the left. Both pupils were equal, round, and reactive to light and accommodation. The movements of the right eye were limited. Evaluations showed exotropia and marked ptosis; hence, based on this evidence and ophthalmology consultation, third and fourth cranial nerve palsy were diagnosed. Anterior segments and intraocular pressure were normal in both eyes. There was no sign of inflammation in the anterior and posterior chambers.

Cardiac, chest, dermatologic, and neurologic examinations were unremarkable.

## 3 Methods

Laboratory tests on the first day of admission demonstrated in Table 1.

**TABLE 1:** Laboratory tests on the first day of admission.

Lab parameter	Day 1
White blood cell (WBC)	8.0 × 1000/mm
Hemoglobin (Hb)	10.9 g/dL
Platelet (PLT)	145 × 1000/mm
Differential leukocyte count	Neutrophils = 80 Lymphocytes = 20
Erythrocyte sedimentation rate (ESR)	16
C‐reactive protein (CRP)	21 mg/L
Creatinine	1.2 mg/dL
Lactate dehydrogenase (LDH)	725 U/L
Cardiac troponin I (high sensitive)	792 ng/L
Aspartate aminotransferase (AST)	61 U/L
Alanine aminotransferase (ALT)	68 U/L
Alkaline phosphatase (AlkP)	246 U/L

**FIGURE 1:**
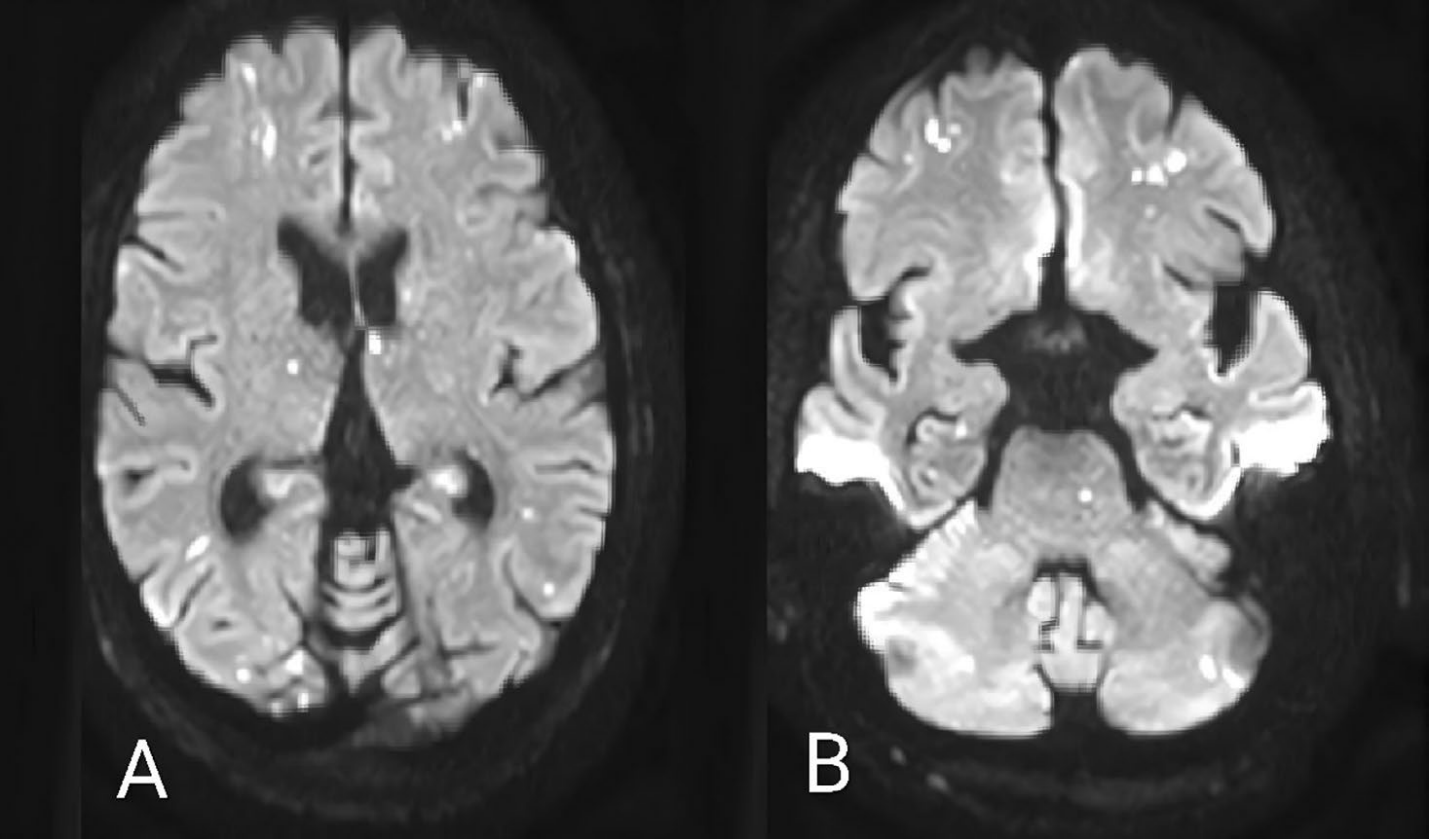
Brain magnetic resonance imaging (A and B) revealed multiple T2‐hyperintensities with restriction, indicative of embolic stroke infarcts affecting cortex, subcortical white matter, caudate nucleus, pons and cerebellum.

On the second day of hospitalization, the patient's tests were repeated; white blood cell count was 13.4 × 1000/mm^3^ with eosinophil cell count 4.8 × 10^9^/L (36.0% of leukocytes), without thrombocytopenia. C‐reactive protein (CRP) level was 21 mg/dL (normally 0–0.35 mg/L). troponin I level was increased to 988 ng/mL (normally 0.0344 ng/mL). Brain MRI was performed for the patient, which was in favor of an embolic stroke; thus, cardiac investigations were highly suggested (Figure 1).

**FIGURE 2:**
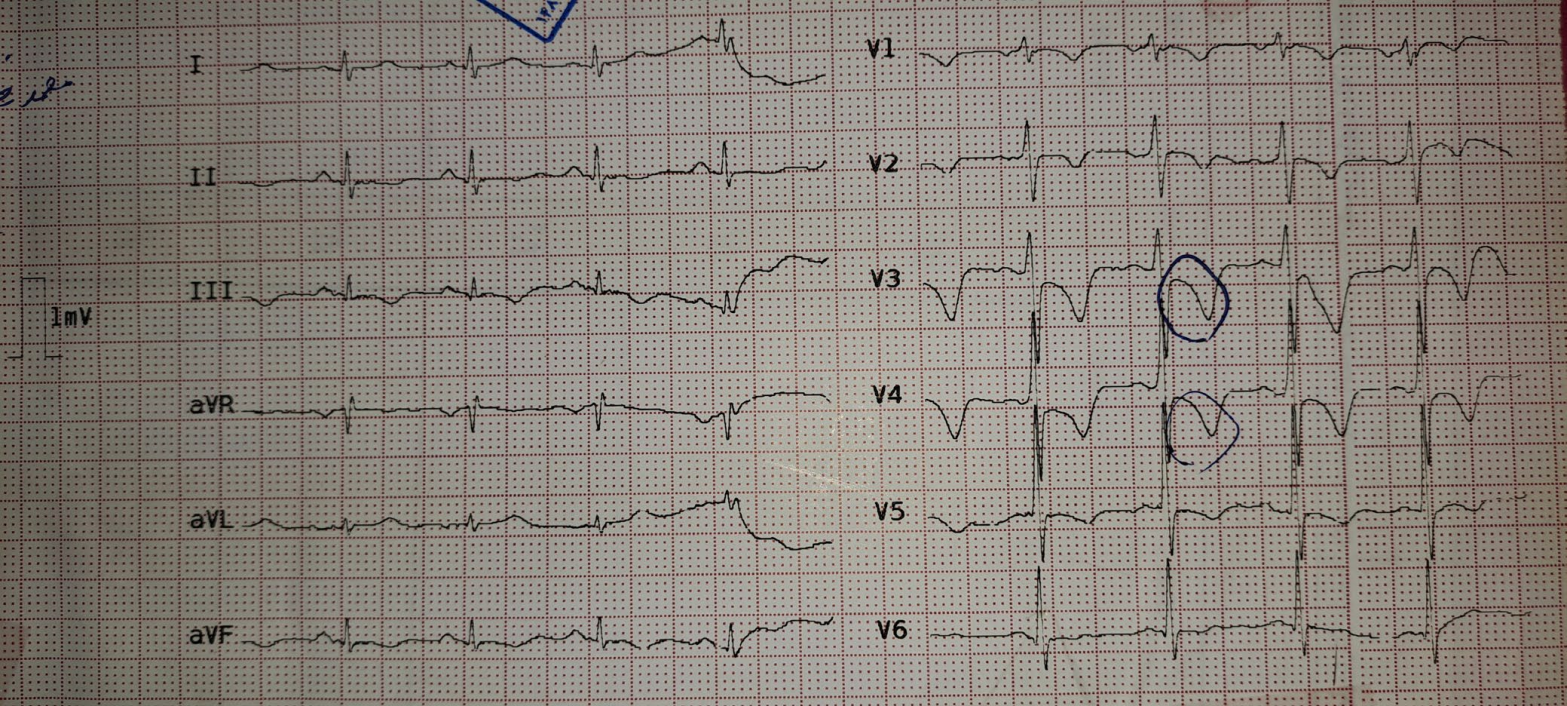
12‐lead electrocardiogram demonstrated ST‐segment depressions and T wave inversions in leads V1‐V5, II, III and aVF.

**FIGURE 3:**
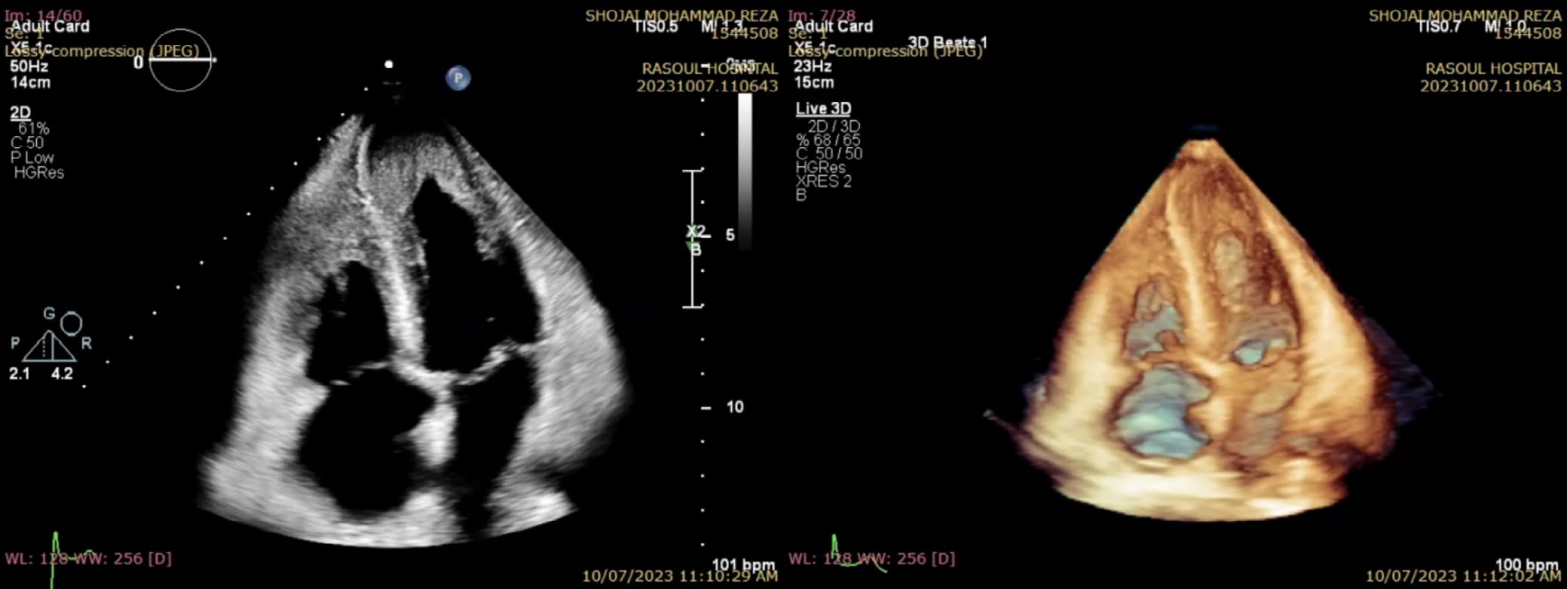
Apical 4‐chamber view of 2D‐TTE (left) and live 3D echocardiography showing obliteration of LV and RV cavities (right).

The patient did not complain of cardiac symptoms. He did not mention the history of chest pain, shortness of breath, and fever. Important ECG findings were T‐wave inversion in precordial and inferior leads. The patient's troponins were 988–792 (Figure 2). Transthoracic echocardiography (TTE) revealed obliterated left ventricular (LV) and right ventricular (RV) apex by echo density (thickness in LV apex: 14 mm, in RV apex: 12 mm) suggestive of hyper eosinophilic syndrome (Figure 3), with a recommendation for cardiovascular magnetic resonance (CMR) for better evaluation of apical hypertrophic cardiomyopathy (HCM). In CMR, there was evidence of faint subendocardial fibrosis in the apical anterior and apical cap and apical RV cavity. CMR findings suggested vasculitis (EGPA).

The next routine blood work showed a white blood cell count of 11.3 × 109/L, eosinophil count of 5.6 × 109/L with a percentage of eosinophils at 50%.

*Further laboratory results*: cytoplasmic‐antineutrophil cytoplasmic antibody (c‐ANCA), perinuclear‐anti‐neutrophil cytoplasmic antibody (p‐ANCA), antinuclear antibodies (ANA), and anti‐double stranded DNA (anti‐dsDNA) were found negative. In addition, erythrocyte sedimentation rate (ESR) was 51 mm/h and C‐reactive protein (CRP) was 32 mg/dL, and Serum IgG4 level was 64 IU/mL (normally 0–100 IU/mL). Lactate dehydrogenase level was 725 IU/L. Lab tests revealed negative results for hepatitis B, hepatitis C, and HIV. Screening for parasites was negative. Blood and urine cultures were sterile. Renal function was normal, and Urinalysis gave normal results.

Bone marrow aspiration was performed and results are as follows: Promyleocyte: 5%, Myelocyte: 11%, Metamyelocyte: 7%, Band: 9%, Segment: 10%, Eosinophi: 40%, Lymphocyte: 6%, Erythroid: 12%, erythroid and myeloid Series: Mature as ordered, Megakaryocytes: adequate and iron stain: 5 in scale (0–6);

Therefore, hypercellular marrow with secondary hyper‐eosinophilia was diagnosed.

## 4 Conclusions and Results

**FIGURE 4:**
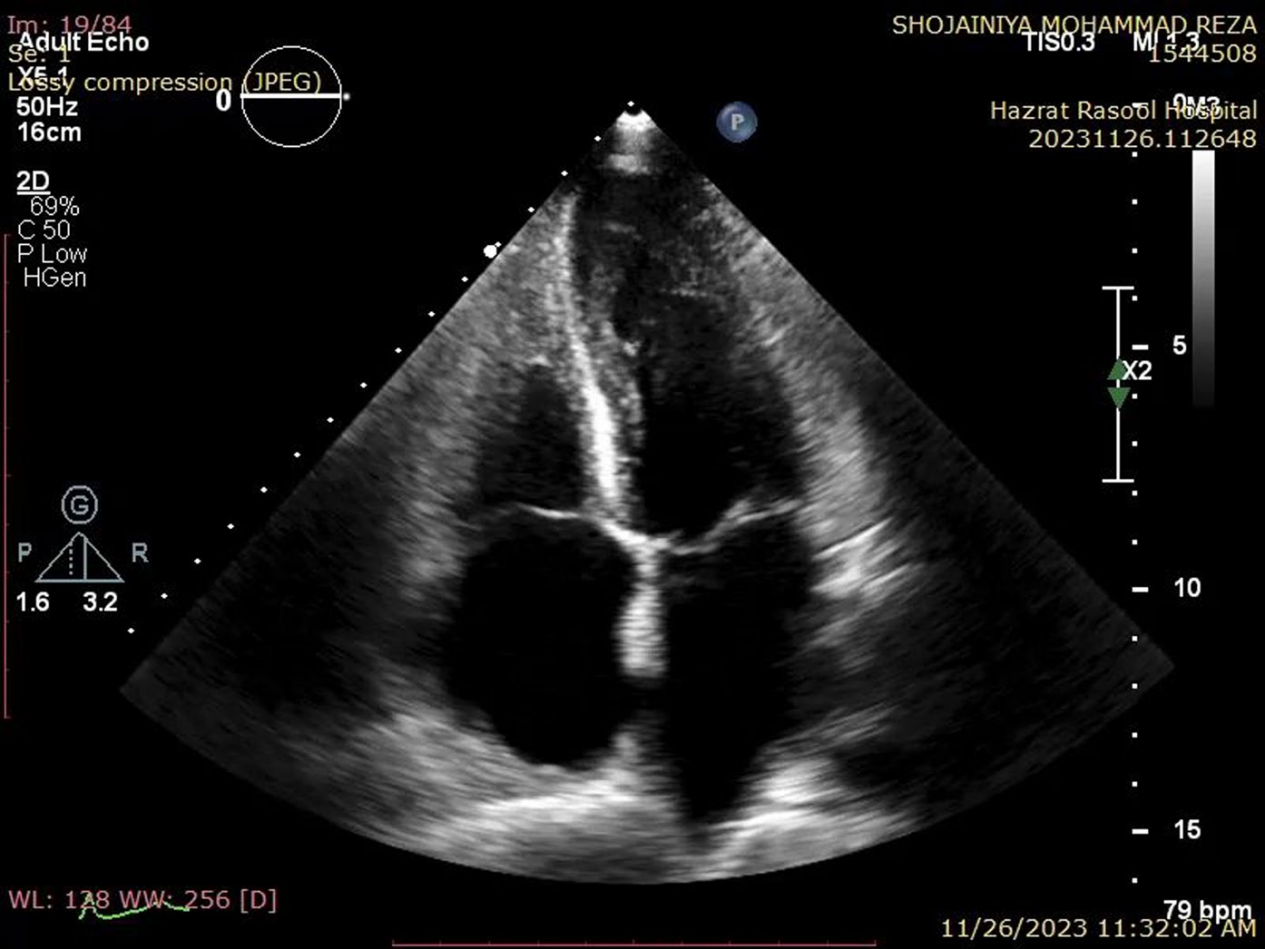
Follow‐up TTE showed no evidence of RV or LV cavity obliteration.

Based on clinical and laboratory findings, the diagnosis of EGPA was made. According to the American College of Rheumatology Criteria, the patient was treated with oral prednisolone 50 mg/day and 1 g of cyclophosphamide. After 7 days, his eosinophil count dropped to a normal level with improvement in eye involvement. The patient is still under follow‐up. Follow‐up TTE showed no evidence of RV or LV cavity obliteration (Figure 4). The response to immunosuppressive therapy was satisfactory, and the patient improved clinically, thus eliminating the need for myocardial biopsy.

## 5 Discussion

This case underscores the diverse clinical presentations of EGPA. The patient presented with neurological deficits, including third and fourth cranial nerve palsy, in conjunction with cardiac abnormalities (subendocardial fibrosis and elevated troponin levels). These findings, combined with significant eosinophilia and a history of asthma, culminated in the final diagnosis of EGPA. The case highlights the need for heightened clinical awareness and a multidisciplinary approach to manage this complex disease.

Neurological involvement is a common feature of EGPA, seen in approximately 60%–70% of cases. Peripheral neuropathy, particularly mononeuritis multiplex, is the most frequently reported manifestation. Central nervous system involvement, including cranial neuropathies, is far less common. The patient's third and fourth cranial nerve palsy, along with ptosis, is a rare presentation, which adds to the complexity of diagnosis. Similar cases have documented ischemic optic neuropathy and orbital pseudotumors as part of EGPA, which reinforces the need to include EGPA in the differential diagnosis of cranial neuropathies in patients with eosinophilia [9, 10, 11].

Cardiac manifestations are among the most critical and life‐threatening complications of EGPA, occurring in 20%–50% of patients. The patient exhibited echocardiographic evidence of obliterated apical segments in the left and right ventricles, with cardiac MRI confirming subendocardial fibrosis, which is a hallmark of eosinophilic myocarditis. Elevated troponin levels further supported active myocardial involvement despite the absence of typical cardiac symptoms like chest pain or dyspnea. This highlights the silent nature of cardiac involvement in some EGPA patients, warranting routine cardiac screening in suspected cases [12, 13].

The diagnosis of EGPA in this patient was challenging, as ANCA antibodies were negative. This finding is seen in approximately 40% of EGPA cases. This underscores the variable manifestations of the disease and the need for clinicians to rely on a combination of clinical criteria, imaging studies, and histopathological findings for accurate diagnosis. Advanced imaging modalities, such as cardiac MRI, play a pivotal role in avoiding invasive procedures like myocardial biopsy [14, 15].

Cardiac complications contribute to 50% of the fatalities linked to the EGPA. The myocardium, pericardium, and endocardium may all be involved. Studies indicate a higher prevalence of heart involvement in individuals negative for ANCA [16]. Untreated EGPA causes significant morbidity and mortality. Studies identified older age at disease onset and heart failure as risk factors for poor prognosis [17].

The patient's rapid response to corticosteroids and cyclophosphamide, with normalization of eosinophil counts and improvement in both neurological and cardiac symptoms, is consistent with current evidence. High‐dose corticosteroids remain the cornerstone of EGPA treatment, with cyclophosphamide reserved for severe or refractory cases. The reversal of cardiac abnormalities on follow‐up echocardiography underscores the efficacy of early and aggressive immunosuppressive therapy [18, 19]. Prognosis of EGPA depends on early diagnosis and treatment. Untreated cardiac involvement significantly increases mortality risk, emphasizing the importance of routine cardiac evaluation in all EGPA patients. Long‐term follow‐up is essential to monitor for disease relapse or progression, as EGPA is known for its relapsing–remitting course [20].

This case highlights several critical points:

1. *Early Recognition*: Prompt recognition of atypical presentations, such as cranial nerve palsies or silent cardiac involvement, can prevent irreversible organ damage.
2. *Role of Advanced Imaging*: Cardiac MRI should be considered a non‐invasive alternative to myocardial biopsy in suspected eosinophilic myocarditis.
3. *Multidisciplinary Approach*: Collaboration between neurologists, cardiologists, and rheumatologists is crucial for comprehensive management.

Further research should focus on developing biomarkers for earlier diagnosis and assessing the long‐term impact of novel immunosuppressive therapies, such as biologics, in managing refractory or relapsing EGPA cases.

## Author Contributions

**Mahshid Talebi‐Taher:** conceptualization, supervision. **Sobhan Mehdipourrabori:** data curation, investigation, writing – original draft. **Soroush Mostafavi:** conceptualization, methodology, writing – original draft, writing – review and editing. **Mahboubeh Pazoki:** conceptualization, supervision, writing – review and editing.

## Ethics Statement

The researchers were committed and adhered to the Ethics Committee of the Iran University of Medical Sciences at all stages.

## Consent

The patient gave written consent to publish images and disease details.

## Conflicts of Interest

The authors declare no conflicts of interest.

## Transparency Declaration

Authors declare that the manuscript is honest, accurate, and transparent. No important aspect of the study is omitted.

## Data Availability

Data available on request from the authors.
